# Multiple Levels of PGC-1α Dysregulation in Heart Failure

**DOI:** 10.3389/fcvm.2020.00002

**Published:** 2020-01-30

**Authors:** Shin-ichi Oka, Amira D. Sabry, Keiko M. Cawley, Junco S. Warren

**Affiliations:** ^1^Department of Cell Biology and Molecular Medicine, Rutgers New Jersey Medical School, Newark, NJ, United States; ^2^Nora Eccles Harrison Cardiovascular Research and Training Institute, University of Utah, Salt Lake City, UT, United States; ^3^Department of Internal Medicine, University of Utah School of Medicine, Salt Lake City, UT, United States; ^4^Institute of Resource Development and Analysis, Kumamoto University, Kumamoto, Japan

**Keywords:** PGC-1α, heart failure, epigenetics, histone methylation, transcriptional control, cardiac metabolism, mitochondria

## Abstract

Metabolic adaption is crucial for the heart to sustain its contractile activity under various physiological and pathological conditions. At the molecular level, the changes in energy demand impinge on the expression of genes encoding for metabolic enzymes. Among the major components of an intricate transcriptional circuitry, peroxisome proliferator-activated receptor γ coactivator 1 alpha (PGC-1α) plays a critical role as a metabolic sensor, which is responsible for the fine-tuning of transcriptional responses to a plethora of stimuli. Cumulative evidence suggests that energetic impairment in heart failure is largely attributed to the dysregulation of PGC-1α. In this review, we summarize recent studies revealing how PGC-1α is regulated by a multitude of mechanisms, operating at different regulatory levels, which include epigenetic regulation, the expression of variants, post-transcriptional inhibition, and post-translational modifications. We further discuss how the PGC-1α regulatory cascade can be impaired in the failing heart.

## Introduction

Peroxisome proliferator-activated receptor γ coactivator 1 alpha (PGC-1α) belongs to a small family of transcriptional coactivators, including PGC-1β and PGC-1-related coactivator (PRC), which possess a common function in mitochondrial physiology. PGC-1α was first identified as a cofactor for the nuclear hormone receptor peroxisome proliferator-activated receptor gamma (PPARγ) in adipocytes required for the adaptive thermogenic responses to lower temperature (1). PGC-1α is expressed in several tissue types and highly expressed in metabolically active tissues, which includes brown fat and skeletal and cardiac muscle. In the heart, PGC-1α is an essential molecule in mitochondrial biogenesis and muscle maturation and shares its role with PGC-1β (2). Cardiac-specific ablation of both PGC-1α and PGC-1β is embryonically lethal due to cardiomyopathy (2).

In the past two decades, our understanding of the mechanisms by which PGC-1α regulates cardiac energetics has significantly advanced. PGC-1α binds to several transcription factors, including PPARγ, PPARα, estrogen-related receptor alpha (ERRα), and nuclear respiratory factor 1 (NRF1) [reviewed in (3)]. This explains how PGC-1α signaling can be amplified to a number of metabolic pathways. Therefore, PGC-1α target genes are primarily determined by the transcription factors that PGC-1α interacts with. Gene expression analysis of PGC-1α knockout mice and transgenic mice that overexpress PGC-1α has revealed PGC-1α target pathways, which include mitochondrial biogenesis, oxidative phosphorylation (OXPHOS), fatty acid β-oxidation (FAO), and glycolysis (4–9). Recent studies showed that PGC-1α also enhances autophagy (10–12), apoptosis (13–15), and aging (11), and activates genes that encode enzymes involved in reactive oxygen species (ROS) detoxification in the brain (9, 16).

**Figure 1:**
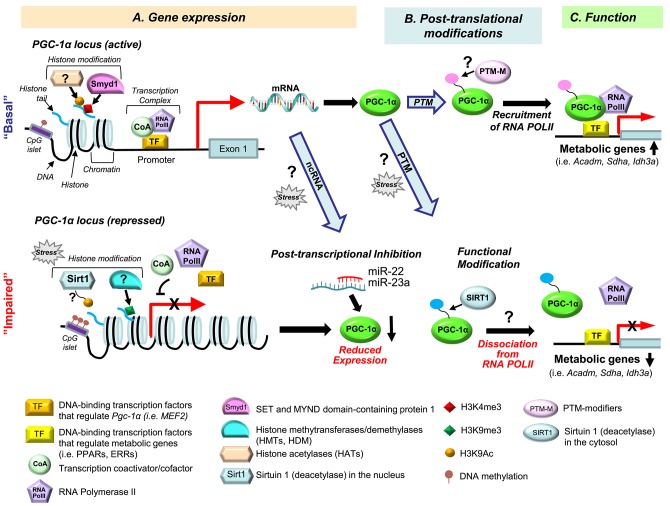
Multiple levels of PGC-1α signaling dysregulation in heart failure. Dysregulation of the PGC-1α regulatory cascade can occur at the level of gene expression **(A)**, post-translational modifications (PTM) **(B)**, and PGC-1α function **(C)**. **(A)** At the gene expression level, the *PGC-1*α cascade can be modulated via histone modifications ((de)methylation and (de)acetylation), DNA (de)methylation, by various transcription factors [TFs, reviewed in (17)], and via post-transcriptional inhibition of the *PGC-1*α gene by non-coding (nc) RNAs. The histone methyltransferase Smyd1 increases promoter activity of *PGC-1*α through regulating the enrichment of the H3K4me3 levels (a gene activation mark) (26). In different animal models of heart failure, reduced expression of PGC-1α was associated with increased H3K9me3 level (a gene repression mark) (27) or a decreased level of H3K9Ac (a gene activation mark) (28). Sirtuin 1 (Sirt1) is a plausible histone deacetylase (HDAC) for gene repression of PGC-1α under pressure overload (28), but this remains to be established. DNA hypermethylation is known to suppress *PGC-1*α in the skeletal muscle (29–31), but little is known about its role in *PGC-1*α regulation in the heart (32). The protein expression level of PGC-1α can be reduced through post-transcriptional inhibition by miRNAs, such as miR-22 (33) and miR-23a (34), but it is unknown whether small ncRNA-mediated PGC-1α repression occurs in heart failure. **(B)** PGC-1α activity is known to be regulated by posttranslational modifications (PTMs), including phosphorylation and acetylation [reviewed in (17)]. However, which PTMs of PGC-1α are specific for the development of heart failure remains unknown. Sirt1 deacetylases the PGC-1α protein (35), but it is not known whether this PTM is a part of PGC-1α dysregulation in the failing heart. **(C)** PGC-1α's transcriptional control of metabolic genes (i.e., *Acadm, Sdha, Idh3a*) largely depends on interaction with DNA-binding transcriptional factors (TF) [i.e., ERRs, PPARs, reviewed in (36)]. In addition, our recent study showed that PGC-1α recruits RNA Polymerase II (RNA PolII) to the promoter regions of PGC-1α target genes (25). Moreover, PGC-1α is dissociated from the promoters of its target genes and RNA PolII in the failing mouse heart (25). We propose that this alteration of PGC-1α behavior in the failing heart is secondary to a PTM of the PGC-1α protein, and intend to test this hypothesis in our future studies.

PGC-1α is a metabolic sensor that enables the body to respond to a plethora of stimuli, including exercise, fasting, and changes in metabolic substrate availability (17). Thus, PGC-1α expression and function are key determinants of energetic states in the heart. Numerous studies have shown that PGC-1α target genes are downregulated in the failing heart (18–20). Some reports have suggested that downregulation of PGC-1α is a major cause of mitochondrial impairment and metabolic defects in the failing heart (7, 8, 21, 22). However, other studies, including ours, suggest that the expression levels of PGC-1α *per se* cannot always explain downregulation of PGC-1α target genes in the failing heart (23–25). In this review, we carefully analyze recent findings in an attempt to construct a holistic picture of the complex mechanisms contributing to impaired PGC-1α regulatory function in the failing heart. We show that these mechanisms operate on multiple levels, including epigenetic and post-transcriptional regulation of PGC-1α expression, as well as altered PGC-1α function occurring under pathophysiological stress (Figure 1). We hope that our analysis helps to identify knowledge gaps in the complex pattern of PGC-1α regulatory network in the heart, and to provide guidance for future studies in this exciting field.

## PGC-1α Expression in Heart Failure

Pathological cardiac hypertrophy is a common response to hypertension, aortic stenosis, and myocardial infarction (37). Transverse aortic constriction (TAC) is a primary animal model for cardiac hypertrophy and heart failure (38, 39). A ligature or a clip is placed across the ascending or descending aorta, leading to the increase of intracardiac pressure (“pressure overload”). TAC initially leads to compensated hypertrophy of the heart, manifested by maintained ejection fraction and temporary enhancement of cardiac contractility (40), in association with metabolic substrate switch from fatty acid to glucose metabolism (41) and a slight increase of glucose oxidation capacity (40). However, over prolonged periods of a chronic hemodynamic overload, an apparently inevitable transition to a decompensated phase takes place, manifested by reduced ejection fraction and cardiac dilation (19, 23, 28, 42). Despite variability of cardiac phenotype in the TAC model (43), most studies using this model have reported energetic abnormalities (19, 20, 23, 44), culminating in a ~30% reduction of myocardial ATP (45), a vitally important physiological constant normally kept within very narrow limits (46). Metabolic remodeling in the setting of pathological cardiac hypertrophy and failure includes decreased myocardial capacity for FAO, reduced ATP production rate, and increased reliance on glucose, concurrent with downregulation of genes that are involved in FAO and mitochondrial oxidative phosphorylation (OXPHOS) (41, 47–50). PGC-1α plays a central role in transcriptional control of those metabolic genes in the heart. PGC-1α knockout mice exhibit deficiencies in cardiac energy reserve and function (7, 21) and the accelerated development of heart failure, in association with downregulation of OXPHOS genes (8). In cultured primary rat neonatal cardiomyocytes, PGC-1α expression was reduced by α1-adrenergic receptor agonist phenylephrine, which recapitulates myocardial remodeling under pressure overload (8). Thus, the decreased expression of PGC-1α has been postulated as an important molecular mechanism for energy starvation and metabolic defects in the failing myocardium. However, the dynamics of PGC-1α expression in the failing heart may be more complex. In animal models of failure, most of the studies showed downregulation of PGC-1α (8, 51–57), but some studies found no change (24, 25, 58). Likewise, analysis of tissue samples obtained from patients at the advanced stage of heart failure showed a variability of outcomes, including decreased gene (59) or protein (60) expression, unchanged gene expression (61, 62), or even a slightly increased gene expression of PGC-1α (63). Of note, in the latter study PGC-1α target genes were coordinately downregulated, underscoring the fact that PGC-1α signaling may be compromised at multiple levels.

The divergent outcomes of these different studies regarding PGC-1α expression in heart failure might be, in part, due to assessment of PGC-1α expression at *different time points* of the disease progression (i.e., early vs. advanced stages, compensated vs. decompensated phases), when PGC-1α expression fluctuates with respect to time, reflecting a combination of adaptive and maladaptive responses to the increased workload. Note that human studies obtain information predominantly from hearts at advanced or terminal stages of heart failure. These stages of the disease are rarely reached in animal studies. Moreover, in human patients with heart failure, PGC-1α expression dynamics may additionally be confounded by different therapeutic interventions (60, 61). Patients with heart failure were treated with various inotropic agents such as β-blockers, diuretics, and angiotensin-converting enzyme (ACE) inhibitors (60). Additionally, human heart failure is pathophysiologically heterogeneous. Depending on the underlying cause, several distinct pathophysiologic conditions, such as ischemia, volume and pressure overload, and metabolic disorders (i.e., diabetes) may contribute to various results of PGC-1α expression. A recent study demonstrates that ischemia triggers distinct epigenetic modifications in heart failure patients (64). Diabetes and obesity are another layer of complexity. In diabetic and prediabetic humans, there is a consistent decrease in the expression of OXPHOS genes that are regulated by PGC-1α and PGC-1β in muscle (65–68). However, cardiac PGC-1α is upregulated in mice that are fed a high-fat diet and in genetically obese (*ob/ob*) mice (69). Thus, it remains unclear how PGC-1α expression is altered in heart failure patients with diabetes and insulin resistance. Differences in age when comparing samples from patients with heart failure and control subjects may also confound results because PGC-1α levels decrease with aging (70). Nevertheless, it is clear that numerous mitochondrial genes and other known targets of PGC-1α, such as glycolytic and FAO genes, are repressed in human heart failure (61, 63), suggesting that dysregulation of PGC-1α may play a role in the pathogenesis of this disease.

Gain and loss of function studies in mice have confirmed the pivotal role of PGC-1α in cardiac energetics (Tables 1, 2). Several gain-of-function models showed increased mitochondrial biogenesis, however, the sarcomeric structure of the heart was disrupted due to uncontrolled mitochondrial proliferation (4, 6). More importantly, those transgenic mice developed dilated cardiomyopathy. In contrast, gain-of-function models with a modest PGC-1α overexpression do not induce heart failure (23, 24) (~3 and ~2 fold at the mRNA level, respectively), suggesting that the development of heart failure in the transgenic mice was due to excessive PGC-1α expression. More importantly, maintaining PGC-1α expression during pressure overload did not show any protective effects on contractile function in this setting (23, 24).

**Table 1:** Cardiac and energetic phenotypes of PGC-1α overexpression mouse models.

**Systemic/Tissue-specific**	**Constitutive/Inducible**	**Cardiac phenotype**	**References**
Cardiac-specific	Constitutive	Uncontrolled mitochondrial proliferation, loss of sarcomeric structure, dilated CM	(4)
Cardiac-specific	Inducible	↑Mitochondrial number and size and upregulation of genes involved in mitochondrial biogenesis during neonatal stages	(6)
		↑Mitochondrial proliferation, derangements of mitochondrial ultrastructure, reversible CM, and ↑venticular mass and chamber dilation in adult mice	
Systemic	Constitutive	↑FAO and cardiac output at baseline and restored expression levels of FAO genes and OXPHOS genes at baseline, no protective effect on TAC-induced cardiac dysfunction, ↑VEGF, and ERRα during pressure overload	(24)
Cardiac and skeletal muscle-specific	Constitutive	No change in cardiac function and energetics with slight decrease in mitochondrial number at baseline, no protective effect on TAC-induced cardiac dysfunction	(23)

*↑: increase, ↓: decrease*.

**Table 2:** Phenotypes of the heart and other organs in PGC-1α knockout mouse models.

**Systemic/Tissue-specific**	**Constitutive/Inducible**	**Cardiac phenotype**	**Effects on other organs**	**References**
Systemic	Constitutive		Constitutively active gluconeogenesis with reduced mitochondrial function in the liver, lean, and resistant to diet-induced obesity due to hyperactivity, lesions in the striatal region of the brain that controls movement	(5)
Systemic	Constitutive	↓Fractional shortening, ↓cardiac performance response to exercise and dobutamine	↓Mitochondrial number and respirator capacity in slow-twitch skeletal muscle with reduced exercise capacity, loss of thermogenic response, ↓oxidative capacity in hepatocytes with hepatic steatosis after short-term starvation, vacuolar lesions in the central nervous system	(21)
Systemic	Constitutive	Normal mitochondrial volume and cardiac function in adult mice (3 months), downregulation of OXPHOS genes, reduced mitochondrial enzymatic activities with energy deficiency (↓ATP, ↓PCr), cardiac dysfunction in old mice (7–8 months)		(7)
Systemic	Constitutive	Normal cardiac function at baseline, accelerated cardiac dysfunction, and chamber dilation under pressure overload		(8)
Systemic	Constitutive		↑Sensitivity to oxidative stress and neurodegeneration in the brain	(9)
Cardiac-specific	Constitutive	Normal cardiac function at baseline, peripartum cardiomyopathy		(71)
Cardiac-specific	Constitutive	Dilated CM, ↓glucose, and fatty acid oxidation, blunted anaerobic metabolism at baseline		(72)
Cardiac-specific	Constitutive	Cardiac hypertrophy and failure, ↓OXPHOS genes, accelerated cardiac dysfunction, accelerated cardiac dysfunction during TAC		(25)

*↑: increase, ↓: decrease*.

In loss-of-function models, two independent lines of global PGC-1α knockout mice were generated. Spiegelman and colleagues showed normal cardiac phenotype and mitochondrial contents under basal conditions (8). However, gene expression analyses revealed upregulation of atrial natriuretic peptide (ANP), brain natriuretic peptide (BNP), and β-MHC, indicative of the presence of cardiac dysfunction (7). The PGC-1α^−/−^ mice generated by the Kelly group exhibited cardiac systolic dysfunction under basal conditions, and cardiac inotropic and chronotropic responses to exercise were both blunted (21). Interestingly, no cardiac dysfunction was observed in those mice when characterized by the other investigators (53). Despite the phenotypic variation in these two lines of global PGC-1α knockout mice, hemodynamic challenge in the form of transverse aortic banding consistently led to pronounced cardiac failure in PGC-1α null mice (8, 53). To further investigate the role of cardiac PGC-1α, three independent groups, including us, have generated cardiac-specific PGC-1α knockout line with identical PGC-1α flox and αMHC-Cre lines (25, 71, 72). The Tavi group and we observed the similar degree of cardiac dysfunction in cardiac-specific PGC-1α knockout mice under basal conditions (25, 72). In contrast, Patten et al. reported normal cardiac function in cardiac-specific PGC-1α knockout mice, but the hearts of female mice exhibited dilated cardiomyopathy after their second delivery (71). Of note, the peripartum cardiomyopathy has not been reported in systemic PGC-1α knockout mice. Taken together, cardiac-specific, rather than systemic PGC-1α knockout mice, prone to develop heart failure. The mechanisms by which the cardiac phenotypes are more pronounced in cardiac-specific PGC-1α knockout mice than systemic knockout mice are currently unknown. Since loss of PGC-1α leads to metabolic derangements in various tissues (Table 2), the complex compensatory mechanisms might take place and mask the effect of PGC-1α deletion on cardiac function.

Overall, the sum of available knowledge strongly suggests that dysregulation of PGC-1α expression is an important factor in cardiac dysfunction and energetic defects in the heart. We will now review recent advances in our understanding of the epigenetic regulation of the *PGC-1*α gene and PGC-1α function.

## Transcriptional Regulation of PGC-1α Gene in the Heart

**Figure 2:**
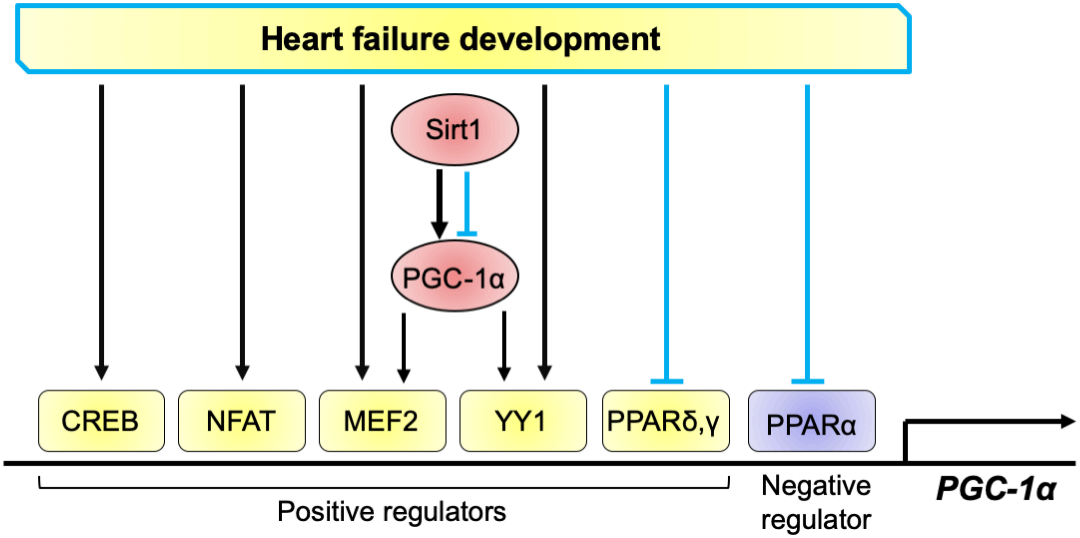
Transcriptional regulation of PGC-1α in the heart. Positive regulators for PGC-1α transcription include CREB, NFAT, MEF2, YY1, PPARδ, and PPARγ, whereas those negatively regulate PGC-1α include PPARα. These factors are activated (black arrows) or inhibited (blue lines) in the progression of heart failure. PGC-1α promotes its transcription through co-activation of MEF2 and YY1. Sirt1 either activates and inhibits PGC-1α, thereby positively and negatively regulates PGC-1α transcription.

Several transcriptional regulators associated with cardiac pathophysiology are involved in transcriptional control of PGC-1α, which include cAMP response element-binding protein (CREB), nuclear factor of activated T-cells (NFAT), myocyte enhancer factor 2 (MEF2), Yin Yang 1 (YY1), PPARs, and Sirt1. Several lines of evidence suggest that some transcription factors that positively regulates PGC-1α transcription are activated or upregulated in the failing heart, such as CREB, NFAT, MEF2, and YY1 (73–75) (Figure 2). The isoforms of PPARs differentially regulate PGC-1α in the healthy and diseased hearts. The mouse proximal PGC-1α promoter contains a typical PPAR response element (PPRE), which is conserved in rat and human (76). PPARδ, but not PPARα, stimulates PGC-1α transcription, although both PPARδ and PPARα bind to the PPRE. PPARγ also stimulates PGC-1α transcription (77). Interestingly, PPARγ-induced PGC-1α transcription is inhibited by PPARα possibly through competition of the binding to PPRE (77). Cardiac-specific PPARα overexpression inhibits PGC-1α transcription (78). Thus, PPARδ and PPARγ positively regulate PGC-1α transcription, whereas PPARα negative regulates the transcription. In the failing heart, PPARα, a negatively regulator for PGC-1α transcription, is inactivated (79). On the other hand, PPARδ and PPARγ, which positively regulate PGC-1α transcription, may also be inactivated, since PPAR target genes involved in fatty acid metabolism are mostly downregulated in the failing heart. Taken together, simultaneous stimulation of the pathways that downregulates and upregulates PGC-1α transcription may be a mechanism responsible for the diverging outcome of PGC-1α expression in the failing heart (Figure 2).

Sirt1 deacetylates lysine residues in both histones and non-histone proteins and regulates the function and transcription of PGC-1α (80). In general, deacetylation of the PGC-1α protein leads to the transactivation of PGC-1α and is known to coactivate PPARα to enhance the gene expression of mitochondrial fatty acid oxidation genes (81). However, Sirt1 can either activate or inhibit PGC-1α through deacetylation in a context dependent manner (35, 82). What determines the PGC-1α function via Sirt1-mediated deacetylation remains unclear. In the heart of systemic Sirt1 knockout mice, PGC-1α is downregulated, indicating that Sirt1 positively regulates PGC-1α (83). However, PGC-1α is also downregulated in cardiac-specific Sirt1 overexpression mouse lines, suggesting that gain of Sirt1 function rather inhibits PGC-1α (84, 85). Whether Sirt1 activates or inhibits PGC-1α in the context of heart failure remains unknown. In the nucleus, Sirt1 acts as an epigenetic modifier and deacetylases histone H3K9/H3K14, leading to chromatin silencing, which occurs at the promoters of myogenin and myosin heavy chain (MHC) in development (86). In our previous study, we demonstrated that Sirt1 deacetylates histone H3K9 in the PGC-1α promoter in the failing heart (28) (Figure 1), which presumably leads to inactivation of the gene. Thus, upregulation of Sirt1 in the failing heart (28) might contribute to the reduced abundancy of PGC-1α.

The transducer of regulated CREB (cAMP response element-binding protein) binding protein (TORC)1, a coactivator of CREB, is another transcription factor that induces PGC-1α, which was identified through screening of 10,000 putative human full-length cDNA in Hela cells for the induction of PGC-1α promoter (87). The other two members of the TORC family, TORC2 and TORC3, also strongly activate PGC-1α transcription. TORC1, 2, and 3 increase the expression of PGC-1α and PGC-1α target genes (Cyt c; CoxII; IDH3α) in mouse primary myotubes (88). In the heart, CREB is activated in response to both physiological and pathological hypertrophic stimuli, which is correlated with upregulation of PGC-1α and increased mitochondrial respiratory rate (89). However, whether TORCs induce PGC-1α and its target genes in the heart needs to be elucidated.

An autoregulatory loop controls PGC-1α expression. The positive feedback loop exists between PGC-1α and MEF2 family of transcription factors: MEF2s bind to the PGC-1α promoter and activate it, predominantly through coactivation by PGC-1α itself (90, 91). This feedback loop allows a stable induction of PGC-1α. It is worth to note that in cardiac-specific PGC-1α knockout mice, the mRNA regions of PGC-1α corresponding to targeted (floxed) exons are significantly downregulated while the other intact regions are rather upregulated (25). This observation suggests that although the autoregulatory transcription loop can enhance PGC-1α induction in response to physiological stimuli, PGC-1α itself might not be essential for PGC-1α transcription.

## Epigenetic Regulation of *PGC-1α* in the Heart

**Figure 3:**
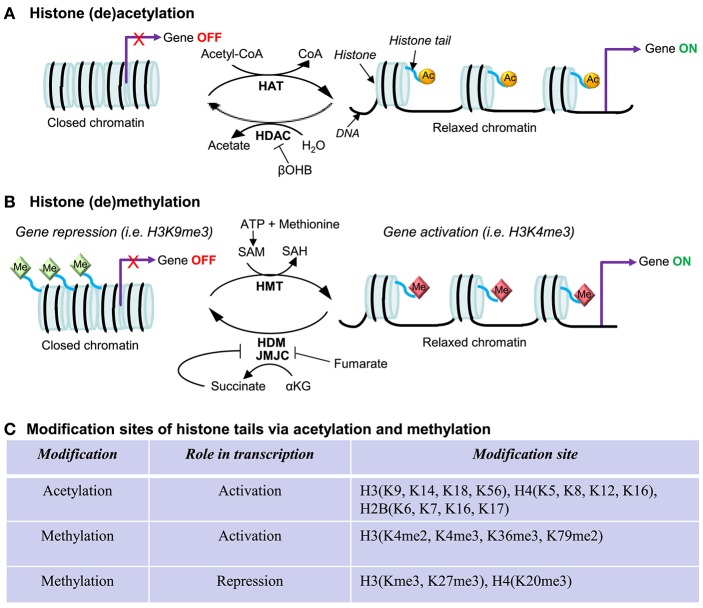
Regulation in gene expression via histone acetylation and methylation. The protruding amino tails of histone proteins can undergo post-translational modifications that affect the expression of genes in close proximity. **(A)** Histone acetylation and deacetylation. Histone lysines are acetylated by histone acetyltransferases (HATs), which use acetyl-CoA as a cosubstrate. Histone deacetylases (HDACs) are grouped in four classes: Classes I, II, and IV are Zn^2+^-dependent and release acetate as a coproduct while sirtuins (class II HDACs) consume NAD^+^ and produce nicotinamide and O-acetyl-ADP-ribose. β-hydroxybutyrate (βOHB) is a ketone body and can inhibit class I and IIa HDACs, being structurally related to be well-known HDAC inhibitor butyrate (95). **(B)** Histone methylation and demethylation. Histones are methylated by histone methyltransferases (HMTs), which require S-adenosylmethionine (SAM) as a consubstrate, yielding S-adenosylhomocysteine (SAH), which is subsequently hydrolyzed to homocysteine and adenosine by SAH-hydrolase (96) Two classes of histone demethylases (HDMs) can remove a methyl group: Lysine-specific demethylase 1 (LSD1) requires the reduction of flavin adenine dinucleotide (FAD) (97), while the Jumonji C (JMJC) domain-containing lysine demethylases catalyze a different demethylation reaction that requires α-ketoglutarate (αKG), O^2^, and Fe(II) (98). Fumarate and succinate, the intermediates in the TCA cycle, are the competitive inhibitors (99, 100). **(C)** Summary of modification sites of histone tails via acetylation and methylation. Other histone post-translational modifications include phosphorylation, ubiquitination, SUMOylation, ADP-ribosylation citrullination, and biotinylation.

Epigenetics refers to reversible modifications of the phenotype without a change in the DNA sequence. In other words, epigenetic regulatory mechanisms can switch genes on or off and determine which proteins are transcribed without changing the inherited genetic program. Epigenetic modifications encompass histone modifications, DNA methylation, and RNA-associated silencing (i.e., microRNAs) (92). The histone landscape is an important part of transcriptional activation (93). The best characterized histone post-translational modifications (PTMs) are acetylation and methylation (94) (also summarized in Figure 3). Histone acetylation is usually associated with gene activation since this process “relaxes” the chromatin allowing for the recruitment of the transcription factors and RNA polymerase (101). This process is mediated by histone acetyltransferases (HATs) and histone deacetylases (HDACs), which add or remove the acetyl groups from histones, respectively. On the other hand, histone methylation is more complex and can occur in various forms: mono- (me), di- (me2), or tri-methylation (me3), with each methylation leading to either gene activation or repression. Histone methylation is catalyzed by histone methyltransferases (HMTs) and histone demethylases (HDMs) (102). Although a large body of work has implicated epigenetic modifications in the development of cardiac disease (102–105), there is a limited number of studies that have examined epigenetic modifications of the *PGC-1*α promoter. Below, we summarize and discuss recent studies reporting histone methylation or acetylation, and DNA methylation in the *PGC-1*α gene. We will also briefly discuss the potential significance of PGC-1α variants, currently well-established in the skeletal muscle but largely unknown in the myocardium.

### Histone Methylation and Acetylation Across *PGC-1α* Promoters

Histone methylation can be associated with either transcriptional repression or activation. For example, trimethylation of histone H3 at lysine 4 (H3K4me3) is an active mark for transcription, while methylation of histone H3 at lysine 9 (H3K9me3) is frequently associated with gene silencing or repression. An excellent overview of histone modifications can be found in Bannister and Kouzarides (93). Our recent study identified the striated muscle-specific histone methyltransferase Smyd1 (SET and MYND domain-containing protein 1) as a novel regulator of PGC-1α in the heart (26) (Figure 1A, top). Smyd1 is known to tri-methylate histone H3K4 (H3K4me3), which generally leads to gene activation (93). Bioinformatic analysis of the heart from the cardiac-specific Smyd1 knockout mice revealed that OXPHOS and the TCA cycle were the most perturbed biological pathways, concomitant with downregulation of the key metabolic regulators PGC-1α, PPARα and RXRα. Furthermore, knockdown of Smyd1 with siRNAs in neonatal rat ventricular cardiomyocytes led to a significant reduction in PGC-1α expression, without significant changes in gene expression of PPARα and RXRα (26). Overall, these data suggested that PGC-1α is a downstream target of Smyd1. Chromatin immunoprecipitation (ChIP) and luciferase reporter assay confirmed that Smyd1 transcriptionally regulates PGC-1α through modulating the H3K4me3 marks on its promoter region (26). In the hypertrophied mouse heart chromatin-bound Smyd1 is increased, while overexpression of Smyd1 in cardiomyocytes prevents cellular hypertrophy under phenylephrine-induced hypertrophic stress (106). Thus, it is plausible that Smyd1 plays a role in maintaining PGC-1α expression as part of adaptive responses to pathological stress through modulating the H3K4me3 marks on its promoter. To support this notion, the ablation of Smyd1 gene in the adult mouse heart led to fulminant heart failure (26). Of note, Smyd1 also acts as a repressor of genes controlling cell growth (106), suggesting the intriguing possibility that Smyd1 is multifunctional in epigenetic regulation of genes involved in metabolic and structural remodeling of the myocardium in response to chronic hemodynamic stress.

The unique histone methylation marks in the *PGC-1*α locus have also been reported in a rat model of high-salt induced-cardiac hypertrophy and failure. In this model, a downregulation of PGC-1α and the reduced mitochondrial respiration capacity in the failing heart were associated with an elevated level of H3K9me3, a marker of gene repression, on the *PGC-1*α promoter (27) (Figure 1A, bottom). Inhibition of histone H3K9 methyltransferases by chaetocin partially normalized PGC-1α expression and H3K9me3 levels in the *PGC-1*α gene (27), confirming a mechanistic link between H3K9me3 marks and *PGC-1*α expression. However, it remains unknown which specific enzymes are responsible for the elevation of H3K9me3 levels in the *PGC-1*α loci in the failing heart.

As for transcriptional regulation of *PGC-1*α through histone (de)acetylation, we have previously reported that in the TAC mouse model of heart failure, the reduced mRNA level of PGC-1α was associated with a significant decrease in H3 lysine 9 acetylation (H3K9Ac) (Figure 1A) (28). Moreover, the reduced H3K9Ac level on the *PGC-1*α promoter was associated with an increase of the promoter occupancy of the histone deacetylase (HDAC) Sirtuin 1 (Sirt1) (28). This raises a possibility that Sirt1 contributes to gene repression of *PGC-1*α under pressure overload through histone deacetylation of the promoter. However, direct evidence indicating the role of Sirt1, or any other HDACs or HATs, in the histone acetylation marks on the *PGC-1*α gene in the heart is lacking. In rat skeletal muscle, the increased level of histone acetylation at H3 lysine 27 (H3K27Ac) of the *PGC-1*α gene was reported in correlation with transcriptional activation after acute exercise (20 min at a speed of 24 m/min on a rodent treadmill) (107).

In summary, cumulative data suggest that posttranslational modifications of histone proteins across *PGC-1*α promoters occur under physiological stimuli and hemodynamic stress. However, the endeavor to understand regulation of the *PGC-1*α gene through histone modifications has just begun. A comprehensive profiling of histone methylation and acetylation marks on the *PGC-1*α promoter in the healthy and diseased heart would greatly advance our understanding of the mechanisms of transcriptional control on the *PGC-1*α gene.

### DNA Methylation of the *PGC-1α* Gene

DNA methylation also controls transcriptional activity of genes. The addition of a methyl group on position 5 of cytosine of the cluster of CpG island (a promoter of the regulatory region of genes) is typically associated with a closed chromatin state and leads to gene silencing, which can be passed to the next generation (108).

Little is known about DNA methylation of the *PGC-1*α gene in the heart. Bisphenol A-induced cardiomyopathy caused hypermethylation on the PGC-1α gene, in association with downregulation of PGC-1α (32). More information can be found in studies concerning other organs and tissues. In the skeletal muscle from patients of type 2 diabetes mellitus (T2DM), hypermethylation of the *PGC-1*α gene was observed at cytosine residues (non-CpG nucleotides), which was associated with a reduction in mRNA levels of PGC-1α and mitochondrial DNA (29). The correlation between DNA hypermethylation of the *PGC-1*α promoter and reduced insulin secretion was also demonstrated in pancreatic islet cells from patients with T2DM (30). Moreover, it has been reported that diet can also alter the DNA methylation profile in *PGC-1*α in skeletal muscle. High-fat diet in mice leads to the increase in DNA methylation in *PGC-1*α at −260 nucleotide site in skeletal muscle, concurrent with the reduced expression of total PGC-1α, which was prevented by supplement of bioflavonoid quercetin and quercetin-rich red onion extract (31). Another group showed that quercetin attenuates high-cholesterol-induced cardiac diastolic dysfunction and cholesterol accumulation in rats, in association with the preserved expression level of PGC-1α and the reduced oxidative stress (109). Exercise-induced activation of PGC-1α in the skeletal muscle was associated with DNA hydroxymethylation (110).

Taken together, these studies suggest that DNA methylation of the *PGC-1*α gene may be a general mechanism regulating PGC-1α expression in response to pathophysiological and dietary stimuli. If this is the case, DNA methylation might also play a role in modulation of *PGC-1*α gene expression in the failing heart, but this needs to be determined in future experiments.

### Epigenetics and Mitochondria

Mitochondria are the essential source of epigenetic modifiers. There is a growing awareness that central components of intermediary metabolism in mitochondria are cofactors or cosubstrates of chromatin-modifying enzymes (111) (Figure 3). The concentrations of those metabolic intermediates constitute a potential regulatory interface between the metabolic and chromatin states. In histone acetylation, S-adenosylmethionine (SAM) is the methyl group donor for both histone methyltransferases (HMTs) and DNA methyltransferases (DNMTs), which is generated from methionine and ATP in mitochondria (Figure 3B). Two classes of histone demethylases (HDMs) can remove a methyl group: lysine-specific demethylase 1 (LSD1) requires the reduction of flavin adenine dinucleotide (FAD) (97), and the Jumonji C (JMJC) domain-containing lysine demethylases catalyze a different demethylation reaction that requires α-ketoglutarate (αKG) (98). Fumarate and succinate, the intermediates in the TCA cycle, are the competitive inhibitors of HDMs (99, 100). In histone methylation, acetyl-CoA is used as an acetyl group donor by histone acetyltransferases (HAT), which is also formed in mitochondria from glycolysis or from fatty acid oxidation. β-hydroxybutyrate (β-OHB), a ketone body, can inhibit class I and IIa HDACs, being structurally related to the well-known HDAC inhibitor butyrate (Figure 3A). Both caloric restriction of mice and direct administration of βOHB resulted in enhanced global histone acetylation (95), consistent with decreased HDAC activity. Thus, the activity of central chromatin-modifying enzymes is closely linked to changes in the levels of the metabolites/intermediates in mitochondria.

Recent developments suggest that mitochondrial protein lysine acetylation (LysAc) modulates the sensitivity of the heart to stress and is involved in mitochondrial dysfunction and the development of heart failure [reviewed in (112)]. Myocardial acetylproteomics revealed that extensive mitochondrial protein lysine hyperacetylation occurs in the early stages of heart failure in the mouse TAC heart and in end-stage failing human heart, in association with reduced catalytic function in succinate dehydrogenase A and complex II-derived respiration (113), suggesting the role of LysAc in mitochondrial dysfunction as the primary metabolic remodeling of heart failure. Protein LysAc occurs when an acetyl group is added to a lysine residue by non-enzymatic chemical modification with acetyl-CoA, or by enzymatic acetylation with acetyltransferases, while removal of the acetyl group from lysine requires NAD^+^ and is mediated by deacetylases, such as sirtuins. Sirtuin 3 (Sirt3) is mainly localized to the mitochondria (114), and loss of Sirt3 in mice leads to the increased mitochondrial LysAc (115). NAD^+^/NADH ratio is the other determinant of energetic states and mitochondrial LysAc, and the elevated NADH/NAD^+^ ratio has been reported in the human failing heart, in association with the increased LysAc levels (116). A recent study demonstrated that increasing myocardial NAD^+^ level via the supplementation of its precursor prevents mitochondrial hyperacetylation and cardiac hypertrophy during pressure overload, concurrent with improved cardiac function (116).

Myocardial contents of the TCA-cycle intermediates (α-KG; fumarate; malate) are decreased in the failing heart, where the mitochondrial capacity of fatty acid oxidation is reduced (117). Interestingly, the changes in metabolome occur earlier than downregulation of OXPHOS genes, suggesting that the regulatory modifications between the metabolic and chromatin states may occur at the early stage of heart failure. Can PGC-1α be involved in this mechanism? Recent study of metabolomic profiling of cardiac-specific PGC-1α knockout mice revealed that cardiac metabolite contents are significantly altered, which includes the decreased level of acetyl-CoA (72), an essential source of epigenetic modifiers (Figure 3). Whether PGC-1α plays a role in the maintenance of the supply for cofactors or cosubstrates of epigenetic modifications needs to be investigated.

### Post-transcriptional Inhibition of PGC-1α Expression

Non-coding RNAs (ncRNAs), which are encoded within the genomes, are generally not translated into proteins. However, ncRNAs play an important role in the regulation of gene expression at the post-transcriptional level. ncRNAs that appear to be involved in epigenetic processes are generally classified into two subgroups based on their length; the long ncRNAs (>200 nt) and the small ncRNAs (<30 nt), the latter of which have three major classes: microRNAs (miRNAs), short interfering RNAs (siRNAs), and piwi-interacting RNAs (piRNAs) [reviewed in (118, 119)]. Among those, miRNAs are the important regulators of gene expression. miRNAs generally bind to a specific target mRNA with a complementary sequence to induce cleavage, degradation, or block translation. It has been estimated that miRNAs are able to modulate up to 60% of protein-coding genes in the human genome at the translational level (120). Thus, they are known to have the potential to fine-tune the expression of numerous genes.

Several miRNAs have been reported to inhibit PGC-1α expression in various organs, which include miR-696 and miR-761 in skeletal muscle (121, 122), miR-199a/214 in brown and beige adipocyte (123), miR29b in cochlear hair cells (124), miR19b/221/222 in vessels (125), and miR485-3p and mi485-5p in breast cancer cells (126). However, very little is known about miRNAs that post-transcriptionally inhibit PGC-1α expression in the heart. miR-23a directly downregulates PGC-1α expression in cardiomyocytes via binding to the 3′UTR of PGC-1α mRNA. Overexpression of miR-23a led to downregulation of PGC-1α and mitochondrial damage in culture cardiomyocytes (34). miR-22 is a muscle-enriched miRNA and post-transcriptionally inhibits PGC-1α as well as PPAR-α and Sirt1 expression (33). Cardiomyocyte-specific overexpression of miR-22 in mice promotes hypertrophic growth and cardiomyopathy, concurrent with downregulation of PGC-1α, PPAR-α, and Sirt1 (33). Whereas, genetic manipulations on miR-23a and miR-22 strongly suggest their involvement in regulation of cardiac metabolism and growth, it remains to be determined whether these or other miRNAs contribute to downregulation of PGC-1α in the failing heart.

### Expression of PGC-1α Variants in the Heart

**Figure 4:**
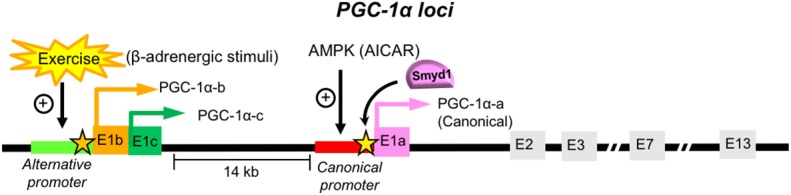
PGC-1α promoters and isoforms. The PGC-1α loci contains two promoters in the skeletal muscle: the *canonical* and *alternative* promoters [Reviewed in (128)]. Transcription initiated from the upstream alternative promoter of the PGC-1α gene results in the inclusion of new exons E1b or E1c, which generate PGC-1α-b and PGC-1α-c, respectively. The PGC-1α-b and PGC-1α-c proteins contain two distinct N-termini, which are different from the canonical PGC-1α-a derived from the exon (E1a) from the canonical promoter. Exercise increases the PGC-1α mRNA levels originated from the *alternative promoter*, which is correlated with the elevated H3K4me3 marks in the *alternative promoter* region of PGC-1α (110) (indicated with an orange star). However, it remains elusive what histone methylation modifiers are responsible for the increase of the H3K4me3 levels on the *alternative* protomer of PGC-1α by exercise. In our previous study, the enrichment of the H3K4me3 marks were assessed in the Smyd1-knockout mouse heart, which was reduced at the canonical promoter (~−1 kb from E1a, indicated with a yellow star), suggesting that Smyd1 regulates the expression of the PGC-1α-a mRNA isoform in the heart. It remains unknown whether the PGC-1α variants from the alternative promoter are involved in metabolic remodeling in the hypertrophied and failing heart.

Transcription of a single *PGC-1*α gene is controlled by multiple promoters coupled to alternative splicing, which give rise to coactivator variants with distinct transcript and protein structure (127). To date, more than ten isoforms of PGC-1α are known to exist, arising from a combination of various promoters and alternative splicing. Currently, two promoters have been identified in the PGC-1α loci of the mouse skeletal muscle: *canonical (proximal)* and *alternative* (Figure 4). The *canonical* promoter originates at exon 1a (E1a), where the canonical PGC-1α-a mRNA isoform and the canonical PGC-1α protein are generated (the 797 amino acid-long murine full-length protein, of which 94.7% of the sequence identifies with the 798 amino acid-long human PGC-1α) (Figure 4). The *alternative* promoter is located ~14 kb upstream of the *proximal promoter*, which is highly conserved between species and has been shown to be active in human skeletal muscle (129, 130). Through alternative splicing, the *alternative* promoter directs the transcription of two different first exons (exon 1b and exon 1c), which generates the PGC-1α-b and PGC-1α-c mRNA isoforms, respectively (130, 131) (Figure 4). The PGC-1α-b and PGC-1α-c proteins differ only in the N-termini while the rest of the protein is identical to the canonical PGC-1α-a. The proteins PGC-1α-a, PGC-1α-b, and PGC-1α-c are all capable in activating PPARs (α, δ, and γ) (132). The combination of these two promoters and splicing provide more variants, such as NT-PGC-1α-1, NT-PGC-1α-c, PGC-1α2, PGC-1α3, and PGC-1α4 (NT-PGC-1αb) [reviewed in (127)].

The PGC-1α gene generates a variety of mRNAs under different biological conditions. Emerging evidence suggests that specific isoforms are induced by physiological stimuli and hypertrophic stress in the skeletal muscle. The mRNA transcripts driven from the *alternative* promoter of PGC-1α were increased by exercise in humans (130, 133, 134) and mice (110), while the mRNA levels of PGC-1α-a driven from the *canonical* promoter remained unchanged in the post-exercised mouse skeletal muscle (132). Interestingly, the protein from the spliced PGC-1α-b from the *alternative* promoter [NT-PGC-1α-b, “PGC-1α-4” in (130)] does not regulate most known PGC-1α targets, such as the mitochondrial OXPHOS, rather it regulates insulin growth factor 1 (IGF1) and myostatin pathways and induces myotube hypertrophy (130). The other study also demonstrated that the administration of β-adrenergic agonist clenbuterol to mice increase the PGC-1α mRNA levels (PGC-1α-b and PGC-1α-c) from the *alternative* promoter without exercise (132). These studies suggest that the expression derived from the *alternative* promoter of PGC-1α is regulated via activation of an β-adrenergic receptor.

Amid this wealth of data obtained from the skeletal muscle, little is known about PGC-1α variants in the heart. One study reported that the mRNA level of a PGC-1α variant NT-PGC-1α is decreased in a mouse model of myocardial infarction (32). It remains unknown whether hemodynamic stress alters the expression of PGC-1α variants in the heart. It is worth to point out that the variability of reported mRNA levels of PGC-1α in the failing heart (8, 23, 24, 55–57), might in part be due to detecting transcripts of different PGC-1α isoforms. Indeed, it is not necessary that all those reported PGC-1α transcripts in the failing heart are the canonical PGC-1α (PGC-1α-a) derived from the *canonical* promoter. For instance, detecting total PGC-1α by targeting exon 2 might mask important changes in the levels of specific isoforms because most variants include the sequence from exon 2. On the other hand, the primers that target the exon 1a will fail to measure the induction or repression of the *alternative* promoter.

What determines the activation and repression of the *alternative* promoters? In other words, what epigenetic modifications regulate the *alternative* PGC-1α promoter? It is possible that the *canonical* and *alternative* promoters are individually regulated by different histone methylation marks and modifiers. Our recent study assessed the H3K4me3 levels at the PGC-1α promoter in Smyd1-knockout mice (26), which is ~1 kb upstream from the *canonical* promoter (Figure 4, indicated as a yellow star). The reduced enrichment of the H3K4me3 by loss of Smyd1 (26) suggests that Smyd1 is likely to regulate the *canonical* promoters. This is consistent with global downregulation of OXPHOS genes (26), which are mainly regulated by PGC-1α-a derived from the *canonical* promoter (128). It remains unknown whether Smyd1 can also methylate the histone proteins within the *alternative* promoter. A recent study of the skeletal muscle showed that exercise leads to the elevation of histone H3K4me3 marks in the *alternative* promoter region of *PGC-1*α, which was correlated with the increases of the PGC-1α mRNA levels originated from the *alternative* promoter (110) (Figure 4, indicated as a red star). However, it remains unknown what histone modifiers are responsible for methylation of the *alternative* promoter in response to exercise.

Summarizing, the expression and function of PGC-1α variants in heart muscle have been somewhat overlooked. However, by analogy with data obtained in the skeletal muscle, it is likely that the profile of PGC-1α isoforms is changing in response to pathological stress, and thus may play a role in adaptive or maladaptive metabolic alterations occurring during the development of heart failure.

## Regulation of PGC-1α Activity by Post-Translational Modifications in the Heart

PTMs, which are equally important as the transcriptional mechanisms, also extensively regulate PGC-1α. To date, phosphorylation, acetylation, ubiquitination, methylation, acetylation, and GlcNAcylation of the PGC-1α protein have been reported. The PTM sites and modulators of the PGC-1α protein are well-described in (17). In particular, phosphorylation of PGC-1α via p38 mitogen-activated protein kinase (MAPK) is clinically relevant. Several diseases, such as heart failure and cancer, cause the elevation of the circulating levels of TNFα and other inflammatory cytokines (i.e., ILα and IL-β) (135), which leads to the nuclear translocalization of p38 MAP kinase, resulting in phosphorylation of PGC-1α at T272, S265, and T298 (136). The phosphorylated PGC-1α via p38 MAP kinase is more stable to degradation and more transcriptionally active, in association with increased mitochondrial respiration capacity and upregulation of OXPHOS genes (136). The expression and activation of p38 MAPK transiently increase in the mouse heart during pressure overload (2 and 4 weeks of TAC) (137). The inhibition of p38 MAPK is beneficial in a mouse model of right ventricular hypertrophy and failure that was induced by pulmonary artery banding (138). It remains elusive whether phosphorylation of PGC-1α via p38 MAPK plays a role in metabolic remodeling in response to hemodynamic stress. Furthermore, which types of PGC-1α's PTMs occur under pathological stress in the heart remains largely unknown. Our previous study demonstrated that the NAD-dependent deacetylase Sirt1 is upregulated in pressure overload-induced heart failure in mice, concurrent with the increased interaction with PPARα (PGC-1α's binding partner), resulting in downregulation of genes involved in OXPHOS and FAO (28). Given that the PGC-1α protein is deacetylated by Sirt1 (35), it is plausible that the upregulation of Sirt1 in the failing heart leads to the deacetylation of PGC-1α (Figure 1C, bottom). The functional consequence of PGC-1α deacetylation in transcriptional control of its target genes and mitochondrial biogenesis is not well-established. In skeletal muscle, most studies have shown that the deacetylation of PGC-1α by Sirt1 increases the co-activation of its target transcription factors (17, 35, 139). However, in one study deacetylation of PGC-1α by Sirt1 did not change exercise-induced mitochondrial biogenesis (140). In the heart, it remains unknown how deacetylation of PGC-1α by Sirt1 modulates its function.

## Multiple Mechanisms by Which PGC-1α Regulates Transcription of Its Target Genes

In the past two decades, our understanding of the role of PGC-1α as a co-activator has significantly advanced. PGC-1α regulates the activity of a large number of transcription factors, including PPARγ (1), PPARα (141), ERRα (142), Forkhead Box O1 (FoxO1) (143), and NRF1 (88). PGC-1α also interacts with p300/CBP, which contains a histone acetyltransferase domain (144), the transcription activator TRAP/Mediator (145), and RNA processing factors (146). Thus, PGC-1α can regulate its target genes via a multitude of mechanisms, which include chromatin modification, preinitiation complex assembly, and RNA processing. Our recent study revealed an additional role of PGC-1α in transcriptional control of its target genes in the heart. We demonstrated that PGC-1α recruits RNA polymerase II (PolII) to the promoters of metabolic genes, which was dissipated in the failing heart (25) (Figure 1C). Chromatin immunoprecipitation-sequencing (ChIP-seq) revealed that the occupancy of PolII to the PGC-1α target gene promoters was consistently reduced in the mouse heart after 4 days of TAC surgery, the time point at which neither mRNA nor protein expression of PGC-1α was changed. ChIP-PCR assays of the mouse failing heart also showed a decreased interaction of PGC-1α with the promoters of its target genes (*Mcad; Sdha; Idh3a; Atp5k1*) in the TAC mouse heart, concurrent with the decrease of PolII's promoter occupancy in those genes (25). In cardiomyocytes, overexpression of PGC-1α induced recruitment of PolII to the promoters of PGC-1α target genes, such as *Mcad* and *Idh3a*. Furthermore, *in-vitro* DNA binding assay using biotin-labeled DNA comprising 380 bp of the *Idh3a* promoter showed that PGC-1α enhances recruitment of PolII to the promoter, whereas siRNA-mediated PGC-1α knockdown inhibits it. Therefore, downregulation of OXPHOS genes in the failing heart is, in part, attributed to the dissociation of PGC-1α from the target gene promoters, rather than the decreased expression levels of PGC-1α. In other words, it appears that pathological stress interferes with the ability of PGC-1α to bind to its target promoters. To support this notion, PGC-1α purified from cardiomyocytes treated with α1-adrenergic agonist phenylephrine had reduced ability to bind to the *Idh3a* promoter, mimicking a pathological consequence of heart failure (25).

It remains unknown what regulates the ability of PGC-1α to recruit PolII to the promoters of metabolic genes. It is plausible the PTMs of the PGC-1α protein occur under pressure overload, which leads to the dissociation of PGC-1α from the target gene promoters (Figure 1C, bottom). Given that Sirt1 was upregulated in the TAC mouse heart, concurrent with downregulation of PGC-1α target genes (28), it is likely that the deacetylation of PGC-1α by Sirt1 is attributed to the dissociation of PGC-1α from its target gene promoters and PolII. To support this notion, less PGC-1α was dissociated from target gene promoters under pressure overload in Sirt1 knockout mice (81). It is our future study to determine the specific PTMs that are responsible for the recruitment of PolII and that interfere with the ability of PGC-1α to bind to its target gene promoters under pathological conditions.

In cardiac-specific PGC-1α knockout mice, where the protein expression was decreased by ~50%, the promoter occupancy of PolII in PGC-1α target genes was decreased, similar to the TAC heart (25). However, maintaining PGC-1α expression during pressure overload by PGC-1α overexpression did not prevent mitochondrial impairment in the TAC mouse heart (24). It is possible that maintaining PGC-1α expression during pressure overload is not sufficient to preserve its function in the recruitment of PolII to the promoters of OXPHOS and FAO genes. The appropriate PTMs of PGC-1α might be required to normalize its function under pathological stress. Therefore, it is critical to determine the specific PTMs and PTM modifiers that are responsible for functional modifications of PGC-1α in the failing heart (Figure 1C).

## Role of PGC-1α in Various Organs

Heart failure is accompanied by a systemic illness that contributes to its progressive nature. Recent studies suggest that heart failure may itself promote systemic metabolic changes such as insulin resistance, in part through neurohumoral activity (147). Moreover, patients with chronic heart failure are characterized by systemic inflammation, as evidenced by elevated circulating levels of several inflammatory cytokines (148). Thus, interorgan cross-talk might contribute to the detrimental self-perpetuating cycle of heart failure (heart failure→altered metabolism in the other organs→heart failure).

**Figure 5:**
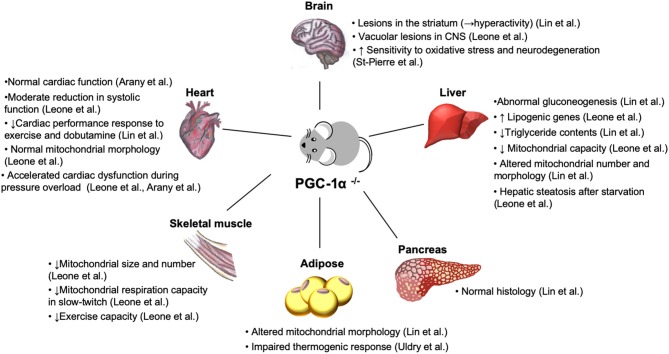
Phenotypes of systemic PGC-1α knockout mice. Loss of PGC-1α leads to impaired energy homeostasis in a variety of organs (5, 21). Despite the reduced density and function of mitochondria in skeletal muscle (21) and abnormality in brown fat tissue with abundant accumulation of large lipid droplets (5), PGC-1α knockout mice are paradoxically lean and resistant to diet-induced obesity due to hyperactivity, resulted from the lesions in the striatum in the brain (5). Normal cardiac function (7, 8) and moderate systolic dysfunction (21) have been reported in two different lines of PGC-1α null mice. Nevertheless, both PGC-1α^−/−^ models show cardiac dysfunction in response to hemodynamic stress and metabolic challenge (8, 21). CNS, central nervous system.

PGC-1α is abundantly expressed in tissues with high energy demand (149). Loss-of-function study in mice suggests that PGC-1α dysfunction leads to multisystem energy metabolic derangements (Table 2, Figure 5). Systemic PGC-1α knockout mice exhibit neurological disorders, in association with the severe lesions in the striatum of the brain area that controls movement (5, 9), which is affected in certain neurogenerative diseases, such as Huntington's disease (150). Similarly, PGC-1α null mice exhibit the accelerated neurodegeneration in response to oxidative stress (9), indicative of the role of PGC-1α in the defense system to ROS. It remains elusive whether the neurological abnormalities in PGC-1α deficiency contribute to the systemic metabolic abnormalities through the alterations in circulating hormones and/or signals that originated from the central nervous system.

Patients with congestive heart failure decrease exercise capacity. Although cardiac dysfunction is the primary pathological insult, emerging evidence suggests that myocardial remodeling in peripheral skeletal muscle occurs independent of cardiac impairment (151). It has been reported that PGC-1α is downregulated in skeletal muscle in heart failure patients (66, 67). Total skeletal muscle PGC-1α deficiency led to a dramatic reduction in exercise performance, concurrent with rapid depletion of muscle glycogen store and mitochondrial biogenic defects (152). In skeletal muscle-specific PGC-1α-KO mice, reduced mitochondrial function and abnormal glucose homeostasis in skeletal muscle led to pancreatic dysfunction in association with the elevated levels of the circulating IL-6 (153). IL-6 treatment of isolated mouse pancreas islet suppresses glucose-stimulated insulin secretion (153), suggesting the cytokine-mediated crosstalk between skeletal muscle and pancreas.

PGC-1α also plays an essential role in hepatic metabolism. In the liver, loss of PGC-1α led to impaired gluconeogenesis, manifested by lacking hormone-stimulated gluconeogenesis and constitutively activated gluconeogenic gene expression that is completely insensitive to normal feeding controls (5). Interestingly, this phenotype was absent in the different line of PGC-1α knockout mice (21). Consistent with altered mitochondrial number and morphology (5), hepatocytes in PGC-1α knockout mice reduced mitochondrial capacity (21), while the genes involved in lipogenic genes were upregulated with the decreased triglyceride contents (21).

Abnormal morphology was also found in brown fat in PGC-1α knockout mice (5). Induction of thermogenic genes was severely reduced in brown adipose tissue of mice lacking PGC-1α, confirming the essential role of PGC-1α in thermogenesis, while loss of PGC-1α did not affect brown fat differentiation (154). Unexpectedly, PGC-1α knockout mice are lean and resistant to diet-induced insulin resistance (5). This is, in part, due to hyperactivity related to the lesions in striatum in the brain, as described above (5).

It remains unknown whether hemodynamic stress directly leads to the alterations in PGC-1α expression in those organs besides cardiac muscle. The cytokine-mediated metabolic changes might be one of the possible mechanisms leading to multisystem metabolic derangements in heart failure.

## Conclusions

In this review, we summarized multiple mechanisms by which the PGC-1α regulatory cascade can be impaired in the failing heart. Whereas, early studies predominantly considered the regulation of PGC-1α transcription, it is now clear that PGC-1α dysregulation may occur at multiple levels, including epigenetic regulation of the *PGC-1*α gene, post-transcriptional inhibition via miRNAs, the expression of PGC-1α variants, and post-translational modifications of the PGC-1α protein. However, at each of these levels, the current knowledge remains limited and many questions remain to be answered. At the epigenetic level, whereas dynamic changes in histone marks across *PGC-1*α promoters have been documented, the factors inducing these changes are largely unknown. We provided evidence that Smyd1 is one of the factors. Our recent work also suggests that Sirt1 may be involved, but its role needs to be directly demonstrated. What other histone modifiers are involved in epigenetic regulation of the *PGC-1*α gene remains to be established.

A recurrent theme of this review is that the cardiac field is lagging behind other fields of science in the understanding of PGC-1α regulation. Whereas, in several organs and tissues DNA methylation of the *PGC-1*α gene has been implicated in response to pathophysiological and dietary stimuli, the prominence of this mechanism in the heart remains to be established. Likewise, the cardiac field is lagging behind in the understanding the role of PGC-1α splice variants in the regulation of the organ (heart) function and metabolism. Studies performed in skeletal muscle suggest that PGC-1α splice variants may regulate disjoint sets of target genes. We cannot exclude a similar arrangement in the heart muscle. One particular motivation to address this issue is the fact that reported variability in PGC-1α expression in the failing heart might result from an indiscriminate detection of different sets of PGC-1α splice variants in different studies—and a finer analysis might reveal that a certain PGC-1α variant is consistently downregulated.

Whether and which types of PGC-1α's PTMs occur under pathological stress in the heart remains largely unknown. We believe, however, that better understanding of PTMs in this context may be a key to explaining downregulation of PGC-1α target genes in those cases when PGC-1α expression is preserved in the failing heart (19, 23, 25, 61, 62). We have proposed a hypothesis that under pathological stress PGC-1α undergoes PTMs which interferes with its ability to recruit polymerase II to the promoters of OXPHOS and FAO genes. We hope to prove this hypothesis in our future studies.

## Author Contributions

All authors listed have made a substantial, direct and intellectual contribution to the work, and approved it for publication.

### Conflict of Interest

The authors declare that the research was conducted in the absence of any commercial or financial relationships that could be construed as a potential conflict of interest. The reviewer, KD, declared a past co-authorship with one of the authors, SO, to the handling editor.
